# Effects of dyslipidemia on E antigen seroconversion of patients with chronic hepatitis B treated by nucleoside (acid) analogs

**DOI:** 10.1186/s12944-021-01582-x

**Published:** 2021-10-30

**Authors:** Ziqiang Xia, Juzeng Zheng, Liang Zheng, Endian Zheng, Zhuolin Zou, Xiong Sheng, Jinming Wu

**Affiliations:** 1 Department of Gastroenterology, Wenzhou People’s Hospital, Wenzhou, 325000 China; 2 Department of Gastroenterology, The First Affiliated Hospital of Wenzhou Medical University, Wenzhou, 325000 China; 3 Department of Infectious Diseases, The First Affiliated Hospital of Jiaxing College, Jiaxing, 314000 China; 4 Department of Infectious Diseases, The First Hospital of Jiaxing, Jiaxing, 314000 China

**Keywords:** Dyslipidemia, Chronic hepatitis B, Nucleoside (acid) analogs, Antiviral effect

## Abstract

**Background:**

The prevalence of dyslipidemia in China is increasing annually. Current studies suggest that dyslipidemia affects the antiviral efficacy of hepatitis C virus (HCV) therapies, while recent studies suggest that serum lipids influence the response rates of chronic hepatitis B (CHB) patients receiving PEGylated interferon-alpha (Peg IFN-α) treatment. However, the role of dyslipidemia in the efficacy of nucleoside (acid) analogues (NAs) in CHB patients remains unclear.

**Methods:**

From January 2010 to December 2013, data from 179 treatment-naive patients with CHB who were hepatitis B e antigen (HBeAg)-positive and had visited the first affiliated hospital of Wenzhou Medical University were assessed. Of these patients, 68 were assigned to the dyslipidemia group (diagnosed with CHB complicated with dyslipidemia) and 111 to the normolipidemic group. The following 3 treatment strategies were performed for all CHB patients over a 5-year period: lamivudine (LAM) plus adefovir dipivoxil (ADV) combination therapy, telbivudine (LdT) monotherapy, and entecavir (ETV) monotherapy. Serum assessments, blood biochemistry, HBV serological markers, HBV DNA before treatment and HBeAg serological conversion and virological responses at different timepoints after treatment were compared between the two groups. Measurement data were compared by τ tests and enumeration data by χ^2^ tests. Correlation analysis was performed using binary logistic regression analysis.

**Results:**

The rates of HBeAg seroconversion in the dyslipidemia group at years 1, 2, 3, and 4 were 10.3, 13.2, 17.6, and 22.1%, respectively, which were not significantly lower than those of the normolipidemic group (11.7, 16.2, 18.0 and 33.3%; χ^2^ = 0.085, 0.293, 0.004, and 2.601, respectively; *Ρ* > 0.05). However, the rates of HBeAg seroconversion in the dyslipidemia group were significantly lower than those in the normolipidemic group at year 5 (27.9% vs. 43.2%, χ^2^ = 4.216, *Ρ* < 0.05). Univariate logistic regression analysis revealed significant differences in group, gender, PTA, ALT, AST, CR, and LDL-C between groups with and without seroconversion. Multivariate regression analysis demonstrated that dyslipidemia (OR = 1.993, *Ρ* = 0.038) and male gender (OR = 2.317, *Ρ* = 0.029) were risk factors associated with HBeAg seroconversion.

**Conclusions:**

During antiviral therapy, dyslipidemia affects HBeAg seroconversion in CHB patients treated with NAs, but does not affect the virological response.

## Introduction

Chronic hepatitis B virus (HBV) infection is known to be a major risk factor for hepatocellular carcinoma (HCC) [1]. As per WHO estimates, 296 million people were living with chronic hepatitis B infection in 2019, and ~ 1.5 million new HBV infections are recorded each year. In 2019, hepatitis B accounted for ~ 820,000 deaths, which were majorly contributed by cirrhosis and hepatocellular carcinoma (primary liver cancer) [2]. Interestingly, China alone accounts for nearly half of the global disease burden for chronic HBV. Despite free administration of the hepatitis B vaccine, ~ 93 million individuals are still infected with chronic HBV in China, which includes ~ 20 million patients with chronic hepatitis B (CHB) [3]. Currently, no effective therapies are available to ensure a complete recovery from HBV infection. Once HBV enters the liver cells, it delivers its covalently closed circular DNA (cccDNA) genome. This cccDNA genome is endowed with a long half-life and self-renewal capacity, which protects it against elimination. In addition to these, the unavailability of direct therapeutics further prevents the elimination of cccDNA. Thus, the persistent existence of cccDNA integrating HBV DNA and impairment of innate and specific immunity make the clearance of chronic HBV infection quite difficult.

HBeAg is a non-structural protein that is encoded by the *pre-C/C* gene. It acts as a serum antigen marker after hepatitis B infection. In particular, HBeAg reflects active replication of hepatitis B virus, with the presence of a solid infectious state. Following HBeAg seroconversion, certain patients exhibit a low replication phase, which is characterized by normal serum ALT concentration and minimal liver histological changes, suggesting slower liver damage [4]. Thus, seroconversion is utilized as the target in HBeAg-positive CHB patients.

Interferon (IFNs) and nucleoside (acid) analogs (NAs) can efficiently inhibit HBV replication and reduce the incidence of liver cirrhosis, hepatic failure, and hepatocellular carcinoma (HCC) [5]. In particular, NAs act via inhibition of reverse transcription of pre-genome RNA and HBV DNA synthesis in the cytoplasm. However, these molecules do not exert any influence on HBV cccDNA directly. In comparison to this, interferon promotes host immune defenses against HBV, however, the precise mechanism of the same remains unknown. Recent studies showed that in comparison to NAs, IFNs promoted the degradation of cccDNA and lead to epigenetic modifications in the transcription of cccDNA [6]. Despite their effective activity, the therapeutic applications of common IFN and PEG IFN are quite limited, and their use is prohibited in patients with uncompensated cirrhosis of the liver, acute exacerbation of chronic hepatitis, autoimmune disease, or mental illness. NAs are widely used in China, but these molecules exhibit low efficacy.

With the advancement of the national economy in China, there has been an annual increase in the prevalence of dyslipidemia in the Chinese population. In general, dyslipidemia refers to an increase in the levels of cholesterol (TC) and/or triglycerides (TG) in the serum. Additionally, it also refers to various dyslipidemia states involving symptoms of low-high density lipoprotein cholesterol (HDL-C) in the blood [7]. So far, lipid metabolism as a consequence of chronic HBV infection has been well characterized [8–10]. One of the previous studies showed that the expression of HBV in transgenic mice altered lipid metabolism, and induced oxidative stress in the liver [9]. In another study, it was reported that HBV infection could induce the expression of genes involved in cholesterol synthesis, and thus promoted cholesterol production [11]. Additionally, HBV X protein has been previously shown to inhibit the secretion of apolipoprotein B (Apo-B), and promote the activity of fatty acid synthetase [12, 13]. In turn, it was reported that dyslipidemia influenced HBV infection, which depended on the presence of cholesterol in the viral envelope [14–16]. Furthermore, an increased body mass index in CHB patients was found to be related to hepatic steatosis [15]. Cholesterol has been previously reported to promote HBV infection [16]. It has been previously shown that dyslipidemia is related to the therapeutic response of patients with chronic hepatitis C towards PEG-IFN [17, 18]. LDL-cholesterol (LDL-C) is known to be a predictor of early and sustained virological response. Recent studies have shown that dyslipidemia affects the efficacy of IFN in patients with CHB [19]. However, very limited information is available regarding the effects of dyslipidemia on the efficacy of NAs in CHB patients. Thus, the present study aimed to retrospectively analyze the effects of dyslipidemia on the antiviral efficacy of NAs in HBeAg-positive CHB patients.

## Methods

### Patient data

The study selected a total of 179 newly treated CHB patients with HBeAg-positive hospitalization in the First Affiliated Hospital of Wenzhou Medical University from January 2010 to December 2013, who were diagnosed as per the guidelines for the prevention and treatment of chronic hepatitis B (2015) [20]. The study was approved by the Ethics Committee of the First Affiliated Hospital of the Wenzhou Medical University, and the informed consent of all patients was obtained.

Inclusion criteria: age ≥ 16 years; serum HBV DNA level ≥ 20,000 IU/mL; HBsAg positive, duration > 6 months; HBeAg-positive and anti-HBe-negative. Exclusive criteria: previously received antiviral, immunomodulatory drugs, or corticosteroid therapy; liver diseases by other causes, elevated ALT caused by non-liver diseases; HCV, HDV, or HIV infection; and decompensated liver diseases history or stage. According to the dyslipidemia complication, patients were categorized into two groups, including 68 subjects with dyslipidemia and 111 without dyslipidemia. The diagnostic criteria for dyslipidemia followed the Prevention Guidelines of Dyslipidemia in Chinese Adults (2016 Revision) [7]. The treatment regimens included oral lamivudine (Heptodin, GlaxoSmithKline Pharmaceutical Co., Ltd. Poznań, Poland) 100 mg qd combined with adefovir dipivoxil (Hepsera, GlaxoSmithKline Pharmaceutical Co., Ltd.) 10 mg qd (*n* = 14); or telbivudine monotherapy 600 mg qd (Sebivo, Beijing Novartis Pharmaceutical Co., Ltd., Beijing, China) (*n* = 78); or entecavir monotherapy (Baraclude, Sino-American Shanghai Squibb Pharmaceutical Co., Ltd., Shanghai, China) 0.5 mg qd (*n* = 87); 5-year of treatment. If the drug was discontinued or other drugs were used during the treatment period, the case was withdrawn from the study.

### Detection indicator

Venous blood was drawn for various blood tests after fasting overnight for 12 h. Lipid indexes, including TC (2.44–5.17 mmol/L), TG (0.4–1.70 mmol/L), LDL-C (2.07–3.10 mmol/L), HDL-C (1.29–1.55 mmol/L), and biochemical indexes including ALT (7–40 U/L), AST (13–35 U/L), ALP (50–135 U/L), and r-GT (7–45 U/L) were measured by using standard techniques. Semi-quantitative analysis of HBV serological marker, including HBsAg and HBeAg, was performed by electrochemiluminescence immunoassay (ECLIA). In addition, the quantitative detection of HBV DNA was performed, and the HBeAg seroconversion rates and the virological response rates from 1 to 5 years of treatment were calculated. HBeAg seroconversion was analyzed by detecting anti-HBE and HBeAg. The standard of virological response was the content of hepatitis B virus nucleic acid < 30 IU/mL, as detected by ABI7500.

### Statistical analysis

SPSS 25.0 statistical software was used for all data analyses. Measurement data are presented as mean ± SD using τ tests. Enumeration data were assessed by the rate utilizing χ2 test, the corrected χ2 test, or Fisher’s exact test. Correlation analysis was performed by using Binary Logistic Regression analysis. *Ρ* < 0.05 indicated statistical significance.

## Results

### Baseline data

The present study involved the assessment of data obtained from 179 HBeAg-positive CHB patients, which included 143 males and 36 females, aged 16–75 years (average age = 40.4 ± 12.0 years). These CHB patients were primarily subjected to treatment either with combination therapy involving lamivudine and adefovir dipivoxil, telbivudine monotherapy, or entecavir monotherapy, for 5 years. In particular, 14 subjects were treated with lamivudine combined with adefovir dipivoxil, 78 subjects were treated with telbivudine monotherapy, and 87 subjects were treated with entecavir monotherapy. Among 179 patients, 17 subjects exhibited hypercholesterolemia, 21 subjects had hypertriglyceridemia, and 30 subjects had mixed hyperlipidemia. As shown in Table 1, MBI, TC, TG, and LDL-C were found to be significantly higher in the patients belonging to the dyslipidemia group, when compared with those without dyslipidemia. However, no significant differences were recorded between the two groups in terms of age, gender, ALT, AST, HBsAg, HBeAg, HBV DNA, treatment regimens, or other indicators.

**Table 1:** Comparison of baseline characteristics between dyslipidemia and normolipidemic groups

Baseline Data	Dyslipidemia Group	Normolipidemic Group	τ/χ^**2**^value	***Ρ***
	68	111		
Male (n, %)	57	86	0.415	0.520
Age (years)	39.5 ± 11.9	40.9 ± 12.2	0.795	0.428
Combined therapy (n, %)	5	9	0.187	0.911
BMI (kg/m^2^)	25.0 ± 3.1	22.4 ± 2.2	5.428	0.000*
WBC (× 10^9^/L)	6.3 ± 2.1	5.6 ± 1.4	2.897	0.004*
NE%	0.6 ± 0.1	0.6 ± 0.1	0.138	0.890
NE(×10^9^/L)	3.7 ± 1.7	3.2 ± 1.2	2.586	0.011*
RBC(× 10^12^/L)	4.8 ± 0.6	4.8 ± 0.5	0.217	0.828
HB (g/L)	147.9 ± 15.7	145.5 ± 14.9	1.048	0.296
RDW (%)	13.4 ± 1.2	13.3 ± 2.8	0.158	0.875
PLT (×10^9^/L)	230.5 ± 78.5	215.2 ± 60.9	1.368	0.174
PT (s)	13.5 ± 0.8	13.4 ± 1	0.715	0.476
PTA (%)	92.2 ± 10.0	92.8 ± 13.3	0.341	0.733
INR	1 ± 0.1	1 ± 0.1	0.180	0.857
F (g/L)	3.1 ± 0.6	3.1 ± 0.9	0.398	0.691
APTT (s)	37.3 ± 4.2	36.8 ± 5.2	0.795	0.428
APTTR	1.0 ± 0.1	1.0 ± 0.2	0.140	0.889
TT (s)	17.1 ± 1.3	17.1 ± 1.5	0.175	0.862
TTR	1.0 ± 0.1	1.0 ± 0.1	0.228	0.820
TB (umol/L)	25.4 ± 58.9	15.1 ± 20.1	1.692	0.092
DBIL (umol/L)	15.3 ± 50.3	7.1 ± 18.1	1.502	0.135
TP (g/L)	75.5 ± 6.2	73.7 ± 6.6	1.889	0.061
A (g/L)	43.9 ± 5.6	44.4 ± 5.3	0.587	0.558
ALT (U/L)	349.1 ± 275.2	297.1 ± 224	1.314	0.191
AST (U/L)	249.1 ± 175	266.7 ± 175.9	0.830	0.408
ALP (U/L)	90 ± 22	84.1 ± 23.2	1.719	0.088
r-GT (U/L)	75.9 ± 56.6	72.5 ± 42	0.430	0.688
FBG (mmol/L)	5.3 ± 1.3	5.4 ± 1.6	0.697	0.487
BUN (mmol/L)	4.8 ± 1.4	4.5 ± 1.1	1.338	0.184
CR (mmol/L)	69.3 ± 12.5	67.6 ± 13.7	0.838	0.403
UA (umol/L)	379.9 ± 105	359.9 ± 99.4	1.257	0.211
TC (mmol/L)	5.5 ± 1.3	4.0 ± 0.7	10.210	0.000*
TG (mmol/L)	2.2 ± 0.8	1.1 ± 0.3	12.633	0.000*
HDL-C (mmol/L)	1.1 ± 0.4	1.1 ± 0.3	0.284	0.777
LDL-C (mmol/L)	3.4 ± 1.0	2.3 ± 0.7	8.574	0.000*
HBsAg S/CO	7557.4 ± 10,826.3	5377.3 ± 7487.7	1.494	0.137
HBeAg S/CO	901.5 ± 514.4	777.3 ± 512.9	1.567	0.119
HBV DNA Log_10_IU/mL	7.4 ± 1.2	7.6 ± 1.1	1.114	0.267

*comparison between the dyslipidemia and normolipidemic groups *P* < 0.05

### HBeAg seroconversion rates after antiviral treatment with NAs

**Fig. 1:**
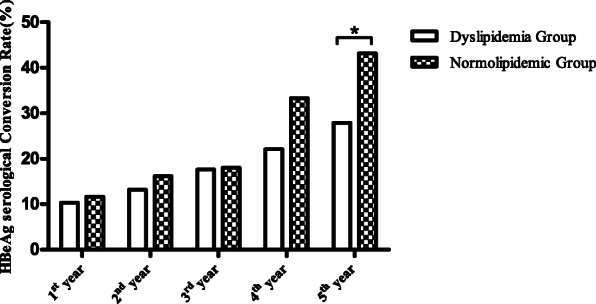
Comparison of the HBeAg serological conversion rates between dyslipidemia and normolipidemic groups at different treatment times. *Comparison between dyslipidemia and normolipidemic groups *P* < 0.05

As shown in Table 2, seroconversion rates for the HBeAg group with dyslipidemia were recorded to be 10.3, 13.2, 17.6, and 22.1% for years 1, 2, 3, and 4, respectively. These values were found to be lower than those reported for normolipidemic group (11.7, 16.2, 18.0, and 33.3%, respectively), however, these differences were not statistically significant (χ^2^ = 0.085, 0.293, 0.004, and 2.601, respectively, *Ρ* > 0.05). For the 5th year, HBeAg seroconversion rates of 27.9 and 43.2% were recorded for the dyslipidemia group and normolipidemic group, respectively. Importantly, these differences were found to be statistically significant (χ^2^ = 4.216, *Ρ* < 0.05) (Fig. 1).

**Table 2:** Comparison of the HBeAg serological conversion rates between the dyslipidemia and normolipidemic groups at different treatment times (number of subjects, %)

Groups	Number of Subjects	HBeAg Serological Conversion Rate				
		1st year	2nd year	3rd year	4th year	5th year
Dyslipidemia Group	68	7 (10.3)	9 (13.2)	12 (17.6)	15 (22.1)	19 (27.9)
Normolipidemic Group	111	13 (11.7)	18 (16.2)	20 (18.0)	37 (33.3)	48 (43.2)
Test value		0.085	0.293	0.004	2.601	4.216
*Р*		0.770	0.589	0.950	0.107	0.040*

*comparison between the dyslipidemia and normolipidemic groups *P* < 0.05

### Virological response rates for NAs after antiviral treatment

**Fig. 2:**
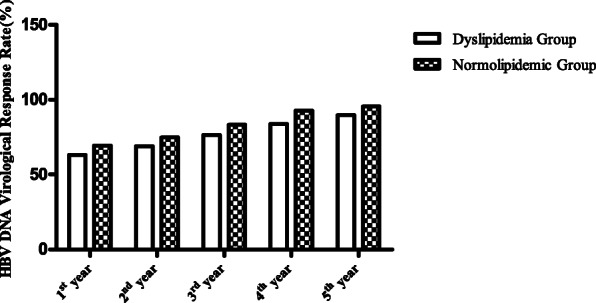
Comparison of HBV DNA virological response rates between dyslipidemia and normolipidemic groups at different treatment times

The virological response rates for HBV DNA in dyslipidemia group were recorded to be 63.2, 69.1, 76.5, 83.8, and 89.7% for years 1, 2, 3, 4, and 5, respectively. These values were lower than the corresponding values for normolipidemic group (69.4, 74.8, 83.8, 92.8, and 95.5%, respectively). However, these differences were not statistically significant (χ^2^ = 0.718, 0.679, 1.466, 3.573, and 1.429, respectively, *Ρ* > 0.05) (Table 3, Fig. 2).

**Table 3:** Comparison of HBV DNA virological response rates between the dyslipidemia and normolipidemic groups at different treatment times (number of subjects, %)

Groups	Number of Subjects	HBV DNA Virological Response Rate				
		1st year	2nd year	3rd year	4th year	5th year
Dyslipidemia Group	68	43 (63.2)	47 (69.1)	52 (76.5)	57 (83.8)	61 (89.7)
Normolipidemic Group	111	77 (69.4)	83 (74.8)	93 (83.3)	103 (92.8)	106 (95.5)
Test value		0.718	0.679	1.466	3.575	1.429
*Р*		0.397	0.410	0.226	0.059	0.232

### Comparison of baseline data, virological response, and HBeAg seroconversion

**Fig. 3:**
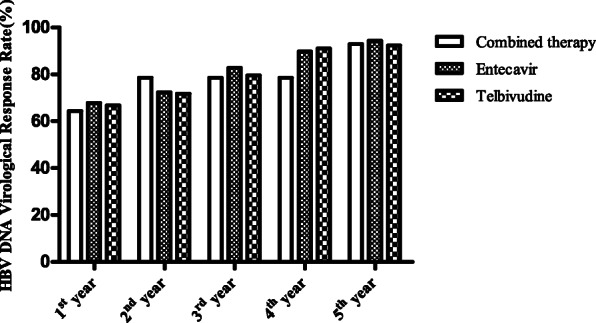
Comparison of the HBV DNA virological response rates after different treatment regimens

**Fig. 4:**
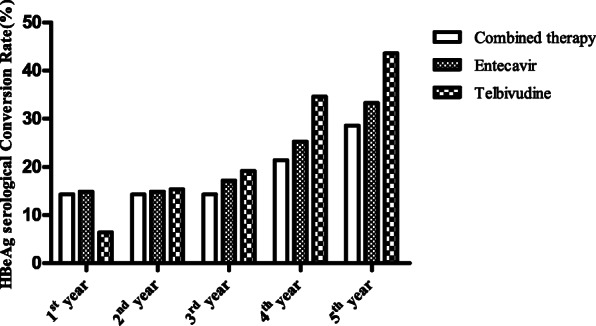
Comparison of the HBeAg serological conversion rates after different treatment regimens

Among 179 subjects, 14 were treated with lamivudine combined with adefovir dipivoxil, 78 were treated with telbivudine monotherapy, while 87 were treated with entecavir. No differences were reported among these three groups in terms of ALT, AST, HBsAg, HBeAg, or HBV-DNA. In addition to these, no significant differences were recorded for HBV DNA virological response rates and HBeAg negative rates for these three groups (Table 4, Figs. 3 and 4).

**Table 4:** Baseline data and comparison of the virological response rates and HBeAg serological conversion after different treatment regimens

Parameters	Combined therapy	Entecavir	Telbivudine	Test Value	***p***
	14	87	78		
Dyslipidemia (n, %)	5 (35.7)	32 (36.8)	31 (39.7)	0.185	0.911
Male (n, %)	12 (85.7)	73 (83.9)	58 (74.4)	2.640	0.267
Age (year)	46.3 ± 11.7	39. ± 10.1	40.4 ± 13.8	2.007	0.138
BMI (kg/m^2^)	23.9 ± 4.8	23.4 ± 2.8	23.4 ± 2.5	0.234	0.792
WBC (×10^9^/L)	5.6 ± 1.7	5.8 ± 1.8	6.0 ± 1.8	0.487	0.615
NE%	0.6 ± 0.1	0.6 ± 0.1	0.6 ± 0.0	0.330	0.720
NE (×10^9^/L)	3.4 ± 1.4	3.3 ± 1.4	3.5 ± 1.4	0.230	0.795
RBC (×10^12^/L)	4.8 ± 0.4	4.8 ± 0.5	4.8 ± 0.6	0.248	0.780
HB (g/L)	142.1 ± 16.3	146.1 ± 13.6	147.5 ± 16.7	0.781	0.460
RDW (%)	13.8 ± 2.1	13.6 ± 3.1	13.0 ± 0.9	1.757	0.176
PLT (×10^9^/L)	221.6 ± 97.1	216.2 ± 68.3	226.3 ± 62.6	0.444	0.642
PT (s)	14.0 ± 1.8	13.4 ± 0.9	13.4 ± 1.0	2.418	0.092
PTA (%)	89.1 ± 22.2	93.1 ± 12.1	92.6 ± 9.6	0.637	0.530
INR	1.1 ± 0.3	1.0 ± 0.1	1.0 ± 0.1	3.547	0.031
F (g/L)	3.2 ± 1.15	3.1 ± 0.8	3.0 ± 0.8	0.373	0.689
APTT (s)	37.5 ± 6.4	37.3 ± 4.2	36.6 ± 5.1	0.498	0.609
APTTR	1.1 ± 0.3	1.0 ± 0.1	1.0 ± 0.1	1.309	0.273
TT (s)	16.8 ± 1.8	17.2 ± 1.4	17.1 ± 1.4	0.477	0.621
TTR	1.0 ± 0.2	1.0 ± 0.1	1.0 ± 0.1	0.059	0.943
TB (umol/L)	58.6 ± 131.3	16.2 ± 15.6	15.1 ± 12.8	1.530	0.465
DBIL (umol/L)	46.1 ± 113.55	7.9 ± 11.8	6.9 ± 11.0	0.841	0.657
TP (g/L)	70.9 ± 8.2	74.6 ± 6.63	74.7 ± 5.9	2.263	0.107
A (g/L)	42.7 ± 7.1	44.3 ± 5.6	44.4 ± 4.9	0.621	0.539
ALT (U/L)	247.4 ± 205.0	346.9 ± 287.6	295.8 ± 193.0	1.509	0.224
AST (U/L)	228.6 ± 130.2	248.1 ± 192.7	221.9 ± 162.4	0.467	0.627
ALP (U/L)	81.4 ± 26.6	88.3 ± 21.8	85.0 ± 23.5	0.805	0.449
R-GT (U/L)	56.6 ± 37.9	78.8 ± 56.6	71.4 ± 37.4	1.492	0.228
FBG (mmol/L)	6.2 ± 2.4	5.4 ± 1.4	5.3 ± 1.3	2.699	0.070
BUN (mmol/L)	4.4 ± 1.5	4.7 ± 1.3	4.6 ± 1.1	0.358	0.699
CR (mmol/L)	62.4 ± 15.9	69.2 ± 11.2	68.3 ± 14.6	1.629	0.199
UA (umol/L)	330.7 ± 143.1	364.4 ± 98.5	377.8 ± 96.2	1.358	0.260
TC (mmol/L)	4.0 ± 1.4	4.6 ± 1.1	4.6 ± 1.2	1.826	0.164
TG (mmol/L)	1.5 ± 0.7	1.5 ± 0.7	1.5 ± 0.8	0.215	0.807
HDL-C (mmol/L)	0.9 ± 0.3	1.1 ± 0.3	1.1 ± 0.3	4.091	0.018*
LDL-C (mmol/L)	2.3 ± 1.1	2.7 ± 0.9	2.8 ± 1.0	1.759	0.175
HBsAg(S/CO)	8269.4 ± 13,647.8	5988.1 ± 9224.3	5921.1 ± 7409.6	0.434	0.649
HBeAg(S/CO)	944.3 ± 549.4	798.9 ± 455.3	832.3 ± 573.9	0.491	0.613
HBVDNA Log_10_IU/mL	7.9 ± 0.8	7.5 ± 1.2	7.5 ± 1.1	0.625	0.537
The first year					
HBV DNA Virological Response Rate	9 (64.3)	59 (67.8)	52 (66.7)	0.076	0.963
HBeAg serological Conversion Rate	2 (14.3)	13 (14.9)	5 (6.4)	3.147	0.207
The second-year					
HBV DNA Virological Response Rate	11 (78.6)	63 (72.4)	56 (71.8)	0.276	0.871
HBeAg serological Conversion Rate	2 (14.3)	13 (14.9)	12 (15.4)	0.014	0.993
The third-year					
HBV DNA Virological Response Rate	11 (78.6)	72 (82.8)	62 (79.5)	0.343	0.843
HBeAg serological Conversion Rate	2 (14.3)	15 (17.2)	15 (19.2)	0.243	0.886
The fourth-year					
HBV DNA Virological Response Rate	11 (78.6)	78 (89.7)	71 (91)	1.943	0.379
HBeAg serological Conversion Rate	3 (21.4)	22 (25.3)	27 (34.6)	2.152	0.341
The fifth-year					
HBV DNA Virological Response Rate	13 (92.9)	82 (94.3)	72 (92.3)	0.252	0.882
HBeAg serological Conversion Rate	4 (28.6)	29 (33.3)	34 (43.6)	2.343	0.310

*variance analysis or nonparametric test in different treatment regimens *P* < 0.05

### Correlation analysis between dyslipidemia and HBeAg seroconversion

The results for single-factor logistic regression analysis reported significant differences between groups with and without seroconversion, particularly in terms of group, gender, PTA, ALT, AST, CR, and LDL-C (OR = 1.965, 2.212, 0.959, 1.003, 1.004, 0.97, and 0.668, respectively; *P* = 0.041, 0.036, 0.004, 0.000, 0.014, and 0.022, respectively, Table 5). The factors influencing HBeAg seroconversion were included in multivariate logistic regression analysis, and the results suggested that dyslipidemia and male gender acted as risk factors for HBeAg seroconversion (OR = 1.993 and 2.317; *P* = 0.038 and 0.029, respectively).

**Table 5:** Single-factor logistic analyses

Baseline data	*P*	OR (95% CI)
group	0.041*	1.965 (1.026–3.761)
Male (n, %)	0.036*	2.212 (1.005–4.639)
Age (years)	0.079	0.977 (0.952–1.003)
Combined therapy (n, %)	0.177	0.647 (0.344–1.271)
BMI (kg/m^2^)	0.272	0.937 (0.834–1.052)
WBC (×10^9^/L)	0.352	0.919 (0.770–1.098)
NE%	0.285	0.189 (0.009–3.999)
NE (×10^9^/L)	0.157	0.846 (0.671–1.067)
RBC (×10^12^/L)	0.671	0.886 (0.508–1.547)
HB (g/L)	0.603	1.005 (0.985–1.026)
RDW (%)	0.240	0.837 (0.622–1.126)
PLT (×10^9^/L)	0.955	1.000 (0.955–1.004)
PT (s)	0.065	1.362 (0.981–1.890)
PTA (%)	0.004*	0.959 (0.932–0.987)
INR	0.732	1.505 (0.145–15.615)
F (g/L)	0.643	0.918 (0.638–1.321)
APTT (s)	0.546	1.020 (0.957–1.086)
APTTR	0.216	3.879 (0.452–33.267)
TT (s)	0.648	1.051 (0.848–1.303)
TTR	0.694	1.925 (0.074–50.085)
TB (umol/L)	0.873	0.999 (0.991–1.007)
DBIL (umol/L)	0.891	0.999 (0.990–1.009)
TP (g/L)	0.450	0.982 (0.937–1.029)
A (g/L)	0.370	0.975 (0.920–1.031)
ALT (U/L)	0.000*	1.003 (1.002–1.003)
AST (U/L)	0.000*	1.004 (1.002–1.006)
ALP (U/L)	0.507	1.004 (0.991–1.018)
r-GT (U/L)	0.135	1.005 (0.998–1.011)
FBG (mmol/L)	0.896	0.986 (0.801–1.214)
BUN (mmol/L)	0.441	0.904 (0.700–1.168)
CR (mmol/L)	0.014*	0.970 (0.946–0.994)
UA (umol/L)	0.635	1.001 (0.998–1.004)
TC (mmol/L)	0.088	0.790 (0.602–1.036)
TG (mmol/L)	0.250	0.780 (0.511–1.191)
HDL-C (mmol/L)	0.769	1.161 (0.428–3.153)
LDL-C (mmol/L)	0.022*	0.668 (0.473–0.944)
HBsAg S/CO	0.979	1.000 (1.000–1.000)
HBeAg S/CO	0.945	1.000 (0.999–1.001)
HBV DNA Log_10_IU/mL	0.061	0.774 (0.592–1.012)

*single-factor logistic regression *P* < 0.05

## Discussion

Chronic HBV infection is one of the major health concerns worldwide, with global infection rates of 3.6%. Among these CHB infected patients, 250–350 million people are positive for HBsAg, which varies according to geographical location [21, 22]. When chronic viral infections are left undiagnosed or untreated, it leads to life-threatening complications such as liver cirrhosis and hepatocellular carcinoma (HCC). Importantly, the availability of a prophylactic vaccine has reduced the emergence of new HBV infections among children aged < 5 years, who are most vulnerable to the development of persistent infections [23]. So far, two therapeutic strategies have been approved for the management of chronic HBV infection, namely nucleoside/nucleotide analogs (NAs) and interferon α (IFN-α)/polyethylene glycol interferon (PEG-IFN) [24].

Over the last three decades, China has witnessed a considerable increase in the prevalence rates of dyslipidemia, which corresponded to the development of the national economy of China [7]. Dyslipidemia is directly related to poor eating habits, lack of exercise, and increased age, and it often leads to chronic kidney diseases, cardiovascular disease, diabetes, fatty liver, and other diseases. Blood lipid metabolism has been previously shown to be influenced by chronic HBV infection [8–10], which in turn influences HBV infection [14–16]. Recent studies suggested that dyslipidemia was related to PEG IFN-α responses in CHB patients [19]. However, limited studies have explored the effects of dyslipidemia on the efficacy of NAs in CHB patients. Therefore, clinical investigations are required to assess the impact of dyslipidemia on the antiviral efficacy of NAs on CHB patients. Consequently, the present study explored the effects of dyslipidemia on the antiviral efficacy of NAs on HBeAg-positive CHB patients. Current treatment guidelines recommend the administration of PEG lFN-α, entecavir, or tenofovir disoproxil as the first choice of treatment for primary patients. However, in some of the patients, the use of telbivudine monotherapy or lamivudine combined with adefovir dipivoxil is maintained as antiviral therapy.

The results of the present study showed that virological response and HBeAg seroconversion rates were lower in the group with dyslipidemia when compared with the subjects included in the normolipidemic group. Specifically, no significant differences were recorded in virological response rates for each time point during the treatment. In addition to this, no statistically significant differences were observed in HBeAg seroconversion rates during the first 4 years of the treatment. For the 5th year, statistically, significant differences were recorded for HBeAg seroconversion rates for the two groups. HBeAg seroconversion is usually associated with progressive reduction of the viral DNA quantification. Thus, it was surprising that HBV DNA was not associated with dyslipidemia status. This might be attributed to association with only late-HBeAg seroconversion. The results for binary logistic regression analysis showed that dyslipidemia and male gender acted as risk factors for HBeAg seroconversion. Dyslipidemia is known to affect human immune function, however, the underlying mechanism for the same remains unknown. Bile acid was previously reported to promote the expression of HBV and weaken the antiviral effects of IFN [25]. Since bile acid originates from cholesterol, it can be proposed that bile acid metabolism in CHB patients is affected by dyslipidemia, which influences antiviral effects. Additionally, metabolic stress caused by dyslipidemia has been shown to induce harmful immune activation, resulting in high levels of CD4 + T cells and lowered HBeAg seroconversion, most probably as a consequence of cellular dysfunction [26, 27].

### Comparisons with other studies and what does the current work add to the existing knowledge

Until date, several studies have been conducted to evaluate the effects of dyslipidemia on infection and treatment of hepatitis B. In terms of novelty, in the present study, the effects of dyslipidemia on the E-antigen seroconversion rate of patients with hepatitis B treated by nucleoside (acid) analogues have been discussed for the first time. As a supplement to the existing knowledge, the present study confirmed that dyslipidemia is an independent risk factor for the reduction of seroconversion rates of HBeAg in patients with hepatitis B treated by NAs.

### Study strength and limitations

This study has several strengths. First, the participants were involved in a long-term follow-up, as a result, this study has high homogeneity and obtained systematic and continuous data. Second, this study provides reference for the clinical treatment of hepatitis B patients with dyslipidemia. However, the present study is associated with certain limitations. The present study was based on retrospective analysis and requires confirmation via clinical prospective studies. Furthermore, the present study assessed only a small number of subjects due to the long follow-up period, potentially introducing study bias.

## Conclusions

Altogether the findings of the present study demonstrated that dyslipidemia influenced HBeAg seroconversion. As such, HBeAg seroconversion rates could be improved by controlling blood lipid levels. Consequently, in the case of CHB patients with dyslipidemia, blood lipid levels should be controlled during antiviral therapy. This could be possibly achieved by including diet regulation, weight management, and temperance. If required, anti-hyperlipidemic drugs might be applied to control lipid levels in the blood. However, liver functions should be monitored in such cases as some of the anti-hyperlipidemic drugs have been shown to exert adverse effects on liver functions. In case of adverse reactions, anti-hyperlipidemic drugs must be stopped. The results for HBeAg seroconversion indicated that the virus was in the low replication stage, and the liver damage was mitigated. Therefore, dyslipidemia was found to be related to the progression of liver damage. These findings suggested that the HBeAg conversion rate could be improved by controlling the blood lipid levels, which could further mitigate liver damage.

Different types of dyslipidemia might lead to different situations. Since bile acid is known to upregulate the expression of HBV genes [25] and cholesterol has been shown to affect the metabolism of bile acid, abnormalities in total cholesterol levels could play a key role in the process of dyslipidemia and thus influence HBeAg seroconversion. Future studies must involve more samples, and the correlation of different types of dyslipidemia with HBeAg seroconversion should be explored. In addition to this, the effects of reduction of blood lipid levels on HBeAg conversion rate in patients with dyslipidemia should be clarified. The control of blood lipid levels is of great significance for CHB patients as it can enhance HBeAg seroconversion rate and mitigate liver damage.

## Data Availability

The datasets generated and/or analysed during the current study are not publicly available due [REASON WHY DATA ARE NOT PUBLIC] but are available from the corresponding author on reasonable request.

## Abbreviations

ADV: Adefovir dipivoxil
Apo-B: Apolipoprotein B
cccDNA: Covalently closed circular DNA
CHB: Chronic hepatitis B
ECLIA: Electrochemiluminescence immunoassay
ETV: Entecavir
HBeAg: Hepatitis B e antigen
HBV: Hepatitis B virus
HCC: Hepatocellular carcinoma
HCV: Hepatitis C virus
HDL-C: High density lipoprotein cholesterol
IFNs: Interferon
LAM: Lamivudine
LdT: Telbivudine
NAs: Nucleoside (acid) analogues
Peg IFN-α: PEGylated interferon-alpha
TC: Cholesterol
TG: Triglyceride
